# A década da nutrição, a política de segurança alimentar e nutricional e as compras públicas da agricultura familiar no Brasil

**DOI:** 10.26633/RPSP.2019.84

**Published:** 2019-12-16

**Authors:** Carmem Priscila Bocchi, Élcio de Souza Magalhães, Lilian Rahal, Patrícia Gentil, Rafaela de Sá Gonçalves

**Affiliations:** 1 Ministério da Agricultura Pecuária e Abastecimento BrasíliaDF Brasil Ministério da Agricultura, Pecuária e Abastecimento, Brasília (DF), Brasil.; 2 Ministério da Cidadania Secretaria Especial de Desenvolvimento Social BrasíliaDF Brasil Ministério da Cidadania, Secretaria Especial de Desenvolvimento Social, Brasília (DF), Brasil.; 3 Consultora independente BrasíliaDF Brasil Consultora independente, Brasília (DF), Brasil.

**Keywords:** Segurança alimentar e nutricional, colaboração intersetorial, alimentação saudável, alimentação escolar, Brasil, Food and nutrition security, intersectoral collaboration, healthy diet, school feeding, Brazil, Seguridad alimentaria y nutricional, colaboración intersectorial, dieta saludable, alimentación escolar, Brasil

## Abstract

A aprovação da Década da Nutrição (2016 a 2025) pelas Nações Unidas partiu da constatação de que as causas que levam à má nutrição são complexas e multidimensionais – a exemplo da situação de pobreza e extrema pobreza e da falta de acesso a uma dieta diversificada e de qualidade, que respeite os hábitos e as culturas alimentares dos diversos povos e países. No Brasil, a agenda da segurança alimentar e nutricional sempre foi conduzida por uma visão integrada do sistema alimentar, expressa no conceito de “segurança alimentar e nutricional”. O objetivo do presente artigo é descrever como a experiência brasileira na estruturação de uma agenda pública nacional de segurança alimentar e nutricional nas duas últimas décadas dialoga e converge com a concepção de nutrição e sistemas alimentares abordada nos documentos que instituem a Década. Para tanto, são abordados aspectos relativos à governança da segurança alimentar e nutricional no Brasil e o papel dos programas de compras públicas da agricultura familiar, com objetivo de incentivar a produção, a comercialização e o acesso a uma alimentação saudável em nível local e propiciar um sistema alimentar mais saudável.

## ABTRACT

The proclamation of the Decade of Action on Nutrition (2016-2025) by the United Nations was prompted by the realization that the causes of malnutrition are complex and multidimensional – as exemplified by situations of poverty and extreme poverty and by the lack of access to a quality, diversified diet that respects the dietary habits and cultures of different peoples and countries. In Brazil, the food and nutrition security agenda has always been guided by an integrated vision of the dietary system, expressed in the concept of “food and nutrition security.” The aim of the present article is to describe how the Brazilian experience structuring a national public agenda on food and nutrition security in the past two decades relates to and converges upon the notion of nutrition and food systems covered in the Decade of Action on Nutrition framework. For that, we discuss aspects relating to the governance of food and nutrition security in Brazil and the role of public purchases from family farming, which are aimed at encouraging production, sales, and access to a healthy diet locally and promoting a healthier food system.

## RESUMEN

El Decenio de las Naciones Unidas de Acción sobre la Nutrición (2016-2025) se aprobó a raíz de que se comprobó que las causas de la malnutrición son complejas y multidimensionales, lo que ejemplifica lo que sucede con la situación de pobreza y extrema pobreza y la falta de acceso a una alimentación diversificada y de calidad, en la cual se respeten los hábitos y las culturas de las diversas sociedades y países. En Brasil, la agenda de seguridad alimentaria y nutricional siempre ha sido impulsada por una visión integrada del sistema alimentario, expresada en el concepto de “seguridad alimentaria y nutricional”. El objetivo del presente artículo es describir cómo se relaciona y converge la experiencia brasileña en la estructuración de una agenda pública nacional de seguridad alimentaria y nutricional en los dos últimos decenios con el concepto de la nutrición y de los sistemas de alimentación abordado en los documentos de creación del Decenio. Para ello, se abordan los aspectos relativos a la gobernanza de la seguridad alimentaria y nutricional en Brasil y el papel de los programas de compras públicas de la agricultura familiar, con el objetivo de incentivar la producción, la comercialización y el acceso a una alimentación saludable a nivel local y de propiciar un sistema de alimentación más saludable.

Em 1º de abril de 2016, a Assembleia Geral das Nações Unidas aprovou uma Resolução proclamando a Década da Nutrição, de 2016 a 2025 (1). A Resolução endossou os compromissos apontados na Declaração de Roma sobre Nutrição (2), principal resultado da Segunda Conferência Internacional de Nutrição (ICN2), ocorrida em novembro de 2014. A Declaração de Roma apontou a complexidade e a multidimensionalidade das causas que levam a todas as formas de má nutrição e apresentou um conjunto de fatores associados, tais como as situações de pobreza e extrema pobreza e a falta de acesso a uma alimentação de qualidade e diversificada, que respeite os hábitos e as culturas alimentares dos diversos povos e países e que seja composta por alimentos saudáveis produzidos de maneira sustentável (2).

A má nutrição se manifesta de várias formas, que vão desde a desnutrição e a deficiência de micronutrientes até o sobrepeso e a obesidade. Além de profundos impactos na saúde das pessoas, a má nutrição traz consequências sociais e econômicas irreparáveis a Estados, indivíduos, famílias e comunidades. Diferentemente de algumas décadas atrás, o espectro dos desafios globais em relação à alimentação e à nutrição não se restringe mais apenas à disponibilidade de alimentos: igualmente importante é o desafio da qualidade do que está disponível para consumo (2).

A Declaração de Roma, ao constatar e abordar as expressões e causas diversas da má nutrição no mundo atual, apresenta uma série de compromissos que devem ser assumidos pelos países, como a erradicação da fome e a prevenção de todas as formas de má nutrição. No âmbito da prevenção, destacam-se: as iniciativas para combater a desnutrição, o baixo peso e o sobrepeso nas crianças com idade até 5 anos e a anemia e a deficiência de micronutrientes em crianças e mulheres; a reversão das tendências de crescimento do sobrepeso e obesidade; e a promoção de sistemas alimentares sustentáveis, baseados em políticas públicas coerentes para guiar desde a produção até o consumo dos alimentos, levando em conta os setores relevantes no fornecimento de alimentos, em consonância com as necessidades nutricionais das pessoas. Enfatizam-se ainda a necessidade de alinhar o tema da nutrição às estratégias nacionais, o fortalecimento das capacidades humanas e institucionais para abordar todas as formas de má nutrição, a cooperação entre países, o desenvolvimento de políticas, programas e iniciativas para garantir uma alimentação saudável ao longo da vida e a criação de ambientes alimentares propícios para escolhas alimentares baseadas em práticas saudáveis (2).

Como aponta a Organização Pan-Americana da Saúde (OPAS), a visão de sistema alimentar, enquanto integração de processos, desde a produção até o consumo de alimentos, demanda a formulação e a implementação de políticas públicas articuladas:

A complexidade dos desafios para o alcance de objetivos essenciais para a atualidade como equidade, erradicação da fome e da pobreza, combate a todas as formas de má nutrição e sustentabilidade, levaram à necessidade da articulação de agendas que historicamente vinham sendo desenvolvidas isoladamente, configurando-se uma oportunidade sem precedentes para o alcance de tais objetivos (3, p.16).

Nesse contexto, o objetivo deste artigo é descrever como a experiência brasileira na estruturação de uma agenda pública nacional de segurança alimentar e nutricional dialoga e converge com a concepção de nutrição e sistemas alimentares abordada nos documentos que instituem a Década da Nutrição.

## A AGENDA DA SEGURANÇA ALIMENTAR E NUTRICIONAL NO BRASIL E A DÉCADA DA NUTRIÇÃO

A agenda brasileira sempre foi conduzida por uma visão integrada do sistema alimentar, expressa no conceito de “segurança alimentar e nutricional”: uma visão que mantém unidos os conceitos de segurança alimentar e de nutrição. O Brasil embasou a sua agenda de segurança alimentar e nutricional no princípio da realização do direito humano à alimentação adequada (DHAA), estabelecido na Constituição brasileira desde 2010, e firmou a governança da segurança alimentar e nutricional como uma agenda de Estado, com leis, decretos, orçamento e sistema de monitoramento definidos (4, 5). Essa governança é operacionalizada por meio do Sistema Nacional de Segurança Alimentar e Nutricional (SISAN), que tem por objetivo assegurar que todas as pessoas que vivem em território nacional estejam livres da fome e, ao mesmo tempo, tenham acesso a uma alimentação de qualidade.

Em 2010, um decreto presidencial estabeleceu as diretrizes da Política Nacional de Segurança Alimentar e Nutricional (PNSAN), operacionalizada por meio de planos quadrienais, com diretrizes, metas, recursos e instrumentos de avaliação e monitoramento, envolvendo diferentes setores de governo e da sociedade. O II Plano Nacional de Segurança Alimentar e Nutricional (PLANSAN 2016-2019), que está em vigência, é resultado de um processo de discussão intersetorial e participativo, fruto da 5ª Conferência Nacional de Segurança Alimentar e Nutricional (6). O II PLANSAN organiza um conjunto de políticas, programas e metas (121 no total), envolvendo ações de 14 ministérios e um orçamento anual estimado em quase 25 bilhões de dólares. É nesse contexto que o Brasil apresentou seus compromissos para a Década de Ação em Nutrição (7).

No âmbito da Década, as 60 recomendações contidas no Plano de Ação da Segunda Conferência Internacional de Nutrição foram agrupadas em seis pilares (8), que dialogam com os nove desafios propostos no II PLANSAN 2016-2019. Dessa forma, durante a 44ª Reunião do Comitê de Segurança Alimentar da Organização das Nações Unidas para a Alimentação e a Agricultura (FAO), o Brasil apresentou compromissos mensuráveis, atingíveis, relevantes e com prazo para a Década de Ação em Nutrição, que são convergentes às metas assumidas no II PLANSAN (7).

O primeiro pilar dos compromissos da Década de Ação em Nutrição ressalta a relação de causalidade entre sistemas alimentares sustentáveis e a promoção de alimentação saudável. Reforça a importância de investimentos e políticas públicas que integrem nutrição, alimentação e agricultura, fortalecendo a produção e o processamento local de alimentos, especialmente por agricultores familiares (9). Diversas metas presentes no desafio 3 do II PLANSAN, como “promover a produção de alimentos saudáveis e sustentáveis, a estruturação da agricultura familiar e o fortalecimento de sistemas de produção de base agroecológica”, relacionam-se às recomendações do primeiro pilar dos compromissos da Década da Nutrição. Esse pilar também dialoga diretamente com o desafio 4 do II PLANSAN: “promover o abastecimento e o acesso regular e permanente da população brasileira à alimentação adequada e saudável”.

## A EXPERIÊNCIA BRASILEIRA COM AS COMPRAS PÚBLICAS DA AGRICULTURA FAMILIAR

Os programas de compras públicas implementados no Brasil funcionam para promover o acesso da população à alimentação adequada e saudável. Esses programas constituem políticas públicas cujo objetivo é incentivar a produção, a comercialização e o acesso a uma alimentação saudável em nível local.

O Programa de Aquisição de Alimentos (PAA) e o Programa Nacional de Alimentação Escolar (PNAE) são os principais instrumentos públicos para a aquisição de produtos oriundos da agricultura familiar no Brasil. Até 2003, quando foi criado o PAA, as políticas públicas voltadas para o fortalecimento da agricultura familiar estavam voltadas para apoiar e financiar a produção de alimentos e inexistiam políticas que garantissem a comercialização da produção. O PAA possui duas finalidades básicas: incentivar a comercialização dos alimentos produzidos pela agricultura familiar e promover o acesso à alimentação adequada e saudável. Para o alcance desses dois objetivos, o Programa compra alimentos produzidos pela agricultura familiar, com dispensa de licitação, e os destina às pessoas em situação de insegurança alimentar e nutricional, residentes, em geral, na própria região onde os alimentos foram produzidos.

**FIGURA 1.**
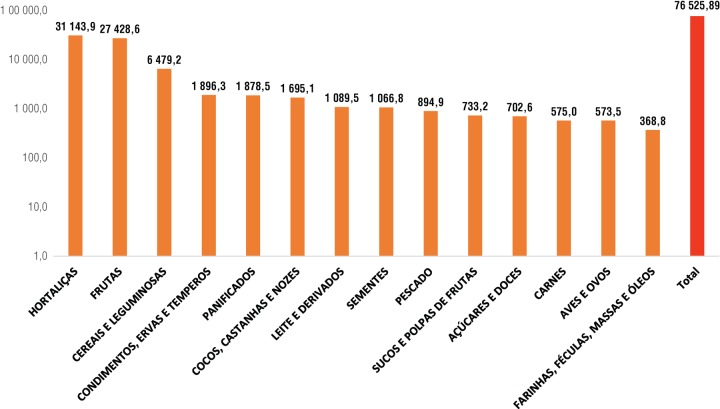
Toneladas de alimentos por grupo doadas pelo governo brasileiro como parte do Programa de Aquisição de Alimentos – Modalidade Compra com Doação Simultânea, 2016

***Fonte***: Ministério da Cidadania, Programa de Aquisição de Alimentos, 2019.

Ao garantir aos pequenos agricultores a aquisição de seus produtos a preços remuneradores, o PAA incentiva-os a planejar e aprimorar sua produção, além de oferecer perspectivas de estabilidade à atividade agrícola, em geral vulnerável a riscos. Ao mesmo tempo, opera como uma política social destinada a garantir que a população em risco alimentar tenha acesso a alimentos de melhor qualidade, na quantidade e com a regularidade necessárias.

Os números da execução do PAA são bastante expressivos: de 2011 a 2017, 4 288 municípios (77% do total de municípios brasileiros) participaram do Programa, tendo sido aplicados R$ 2,87 bilhões (ou aproximadamente 754 milhões de dólares) (10). Vários estudos já descreveram os impactos do PAA sob diversos aspectos. Um relatório de pesquisa publicado pelo Instituto de Pesquisa Econômica Aplicada (IPEA) em 2018 apresentou os resultados de uma revisão sistemática sobre os trabalhos já publicados sobre o Programa. Foram identificados 277 trabalhos, entre monografias, dissertações, teses e artigos de revistas, entre outros. Desses 277, 158 foram selecionados para análise, sendo a maioria estudos de caso em âmbito local e/ou regional, mas com abrangência significativa para todo o Brasil (11). O relatório mostrou uma análise bastante positiva do Programa em várias dimensões:

... os trabalhos demonstram o caráter inovador e estratégico do PAA ao simplificar e agilizar o escoamento da produção, promover e ampliar a inserção socioeconômica dos agricultores familiares, disponibilizar alimentos adequados à nutrição das populações em situação de vulnerabilidade social e insegurança alimentar, habilitar os agricultores familiares para controlar a comercialização de seus produtos, o que resulta no aumento real de suas rendas e no abandono de uma relação de desvantagem com os atravessadores (11, p. 58).

Sobre o PAA, é importante ainda ressaltar a sua capacidade de promover a diversificação da produção, elemento essencial para a promoção de sistemas alimentares mais saudáveis e sustentáveis e para a garantia da segurança alimentar e nutricional (12).

Outro estudo do IPEA que analisou a diversidade dos produtos adquiridos pelo PAA – Modalidade Compra com Doação Simultânea (12) contabilizou, no período de 2011 a 2018, 536 diferentes produtos, resultando num índice de diversidade de Simpson de 0,98, ou seja, bastante significativo. A figura 1 apresenta o volume adquirido de alimentos pelo PAA – Modalidade Compra com Doação Simultânea para o ano de 2016, mostrando a importância do Programa para o consumo de frutas e hortaliças e outros grupos de alimentos considerados saudáveis.

Por sua vez, o PNAE, gerenciado pelo Fundo Nacional de Desenvolvimento da Educação (FNDE), autarquia vinculada ao Ministério da Educação, visa à transferência, em caráter suplementar, de recursos financeiros aos estados, ao Distrito Federal e aos municípios para suprir parte das necessidades nutricionais dos alunos da rede pública de ensino. O PNAE é considerado um dos maiores e mais abrangentes programas na área de alimentação escolar no mundo e vem contribuindo, de forma progressiva, para a realização do DHAA no Brasil. Ao longo dos anos, a concepção do PNAE, que é executado – embora em diferentes formatos – desde a década de 1950, evoluiu de um enfoque assistencialista e de complementação alimentar para uma ótica do direito, conforme preconizado na lei de 2009 que regulamenta o programa. A mesma lei estabelece que no mínimo 30% dos repasses do PNAE deveriam ser utilizados na aquisição de produtos da agricultura familiar (13).

O PNAE atende cerca de 46 milhões de alunos da rede pública de ensino e a compra dos 30% tem se mostrado bastante viável para a maioria dos municípios. Hoje, 86% dos municípios brasileiros adquirem alimentos da agricultura familiar, perfazendo uma média nacional de 22% de compra (14). Assim como o PAA, a compra de alimentos da agricultura familiar pelo PNAE fortalece a comercialização dos alimentos produzidos por esse segmento e, ao mesmo tempo, garante o acesso a alimentos *in natura* e a uma alimentação mais saudável. Promove ainda o crescimento da economia local e do que chamamos de circuitos curtos de produção e comercialização, proporcionando um menor custo dos alimentos e estratégias mais sustentáveis do ponto de vista ambiental (15).

## CONCLUSÕES

O presente artigo apresentou alguns dos compromissos assumidos no âmbito da Década da Nutrição (2016 a 2025) pelo Brasil, que já vinha implementando uma agenda de segurança alimentar e nutricional bem próxima aos conceitos, compromissos e desafios apresentados nos documentos que instituem a Década. Esses desafios estão relacionados à construção de sistemas alimentares mais sustentáveis e saudáveis, caracterizados por ações e políticas públicas que articulem desde a produção até o consumo dos alimentos. Nesse contexto, o PAA e PNAE são exemplos bem-sucedidos de políticas públicas intersetoriais que contribuem para a ampliação do acesso, o fortalecimento da agricultura familiar e a promoção da alimentação saudável.

## Contribuição dos autores.

CPB, ESM, LR, PG e RSG, conceberam a ideia original e levantaram as informações para o artigo. Todos os autores redigiram o artigo, revisaram criticamente o conteúdo e revisaram e aprovaram a versão final.

## Declaração.

As opiniões expressas no manuscrito são de responsabilidade exclusiva dos autores e não refletem necessariamente a opinião ou política da RPSP/PAJPH ou da Organização Pan-Americana da Saúde (OPAS).
